# Kinase Domain Is a Dynamic Hub for Driving LRRK2 Allostery

**DOI:** 10.3389/fnmol.2020.538219

**Published:** 2020-10-06

**Authors:** Susan S. Taylor, Pallavi Kaila-Sharma, Jui-Hung Weng, Phillip Aoto, Sven H. Schmidt, Stefan Knapp, Sebastian Mathea, Friedrich W. Herberg

**Affiliations:** ^1^Department of Pharmacology, University of California, San Diego, San Diego, CA, United States; ^2^Department of Chemistry and Biochemistry, University of California, San Diego, San Diego, CA, United States; ^3^Department of Biochemistry, Institute for Biology, University of Kassel, Kassel, Germany; ^4^Institute of Pharmaceutical Chemistry, Goethe-University Frankfurt, Frankfurt, Germany; ^5^Structural Genomics Consortium, Buchmann Institute for Molecular Life Sciences (BMLS), Goethe-University Frankfurt, Frankfurt, Germany

**Keywords:** protein kinase (PK), GTPase, allostery, hydrophobic cores, Walker motifs, leucin rich repeat kinase 2 (LRRK2)

## Abstract

Protein kinases and GTPases are the two major molecular switches that regulate much of biology, and both of these domains are embedded within the large multi-domain Leucine-Rich Repeat Kinase 2 (LRRK2). Mutations in LRRK2 are the most common cause of familial Parkinson’s disease (PD) and are also implicated in Crohn’s disease. The recent Cryo-Electron Microscopy (Cryo-EM) structure of the four C-terminal domains [ROC COR KIN WD40 (RCKW)] of LRRK2 includes both of the catalytic domains. Although the important allosteric N-terminal domains are missing in the Cryo-EM structure this structure allows us to not only explore the conserved features of the kinase domain, which is trapped in an inactive and open conformation but also to observe the direct allosteric cross-talk between the two domains. To define the unique features of the kinase domain and to better understand the dynamic switch mechanism that allows LRRK2 to toggle between its inactive and active conformations, we have compared the LRRK2 kinase domain to Src, BRaf, and PKA. We also compare and contrast the two canonical glycine-rich loop motifs in LRRK2 that anchor the nucleotide: the G-Loop in protein kinases that anchors ATP and the P-Loop in GTPases that anchors GTP. The RCKW structure also provides a template for the cross-talk between the kinase and GTPase domains and brings new mechanistic insights into the physiological function of LRRK2 and how the kinase domain, along with key phosphorylation sites, can serve as an allosteric hub for mediating conformational changes.

## Introduction

**Figure 1:**
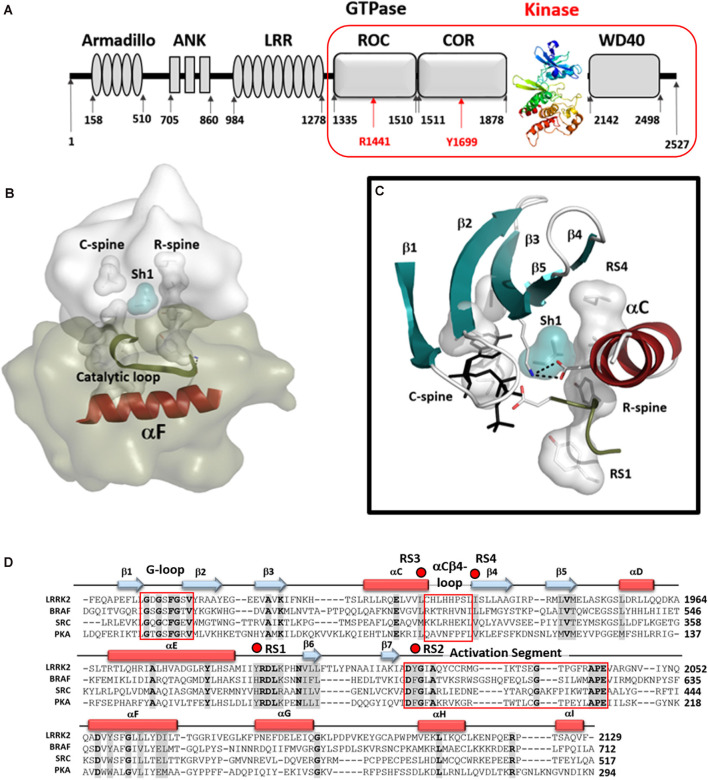
The Kinase domain of Leucine-Rich Repeat Kinase 2 (LRRK2). **(A)** Organization of the domains of LRRK2 with the kinase domain shown as a ribbon colored from blue at the N-terminus to red at the C-terminus. The Cryo-Electron Microscopy (Cryo-EM) structure of the four C-terminal domains [ROC COR KIN WD40 (RCKW)] construct is indicated by a red box. **(B)** A model of the active conformation of LRRK2 is shown where the R-spine residues are aligned into an active conformation and both spines as well as the Catalytic Loop are anchored onto the hydrophobic αF-helix. **(C)** Organization of the N-Lobe in active LRRK2. The five-stranded β-sheet is in teal and the αC-Helix in red. R- and C-spine residues are indicated as shells and the Shell residue (Sh1) that bridges both spines are in teal. The salt bridge between the conserved Lysine in β3 and conserved Glutamic acid in the C-helix is also shown. **(D)** The sequences of the kinase domains of LRRK2, BRaf, Src, and PKA are aligned and the regions corresponding to the G-Loop, the αC-β4 Loop, and the activation segment are highlighted (red boxes). R-spine residues are indicated by red dots. The cartoon indicates the position of β-strands (teal arrows) and α-helices (red rectangles).

The Leucine-Rich Repeat Kinase 2 (LRRK2) is a large multi-domain kinase that is linked through numerous mutations to Parkinson’s disease (PD; Funayama et al., 2002; Paisán-Ruíz et al., 2004; Zimprich et al., 2004; Tan and Skipper, 2007) but is also implicated in Crohn’s disease (Hui et al., 2018). The three N-terminal domains (Armadillo/ARM, Ankryn/ANK, Leucine-rich Repeat/LRR) are classic scaffolds while the four globular and well-folded C-terminal domains (Ras Of Complex/ROC, C-terminal of ROC/COR, Kinase/KIN, and WD40) include the two catalytic domains, the ROC-GTPase, and the kinase. In this manuscript, we refer to the four C-terminal domains (ROC COR KIN WD40) as RCKW (Figures 1A,B). While there are countless examples of cross-talk between kinases and GTPases, LRRK2 is one of the few cases where the kinase and the GTPase domains are embedded within the same polypeptide chain. The GTPase domain of LRRK2 belongs to the Roco protein family and plays an important role as an allosteric effector domain (Bosgraaf and Van Haastert, 2003; Marín et al., 2008). While much information has been gleaned from the PD mutations and the evolutionary precursors of the ROC:COR and ANK:ROC:COR domains from *Dictyostelium* and *C. tepidium*, respectively (Gilsbach et al., 2012; Deyaert et al., 2019; Wauters et al., 2019), high-resolution structural data for human LRRK2 has been largely missing. The first high-resolution human structure came from the Roc domain in 2008 (Deng et al., 2008) but it took 11 years until the next LRRK2 associated structures were published, an extended ROC-domain (Wu et al., 2019), the WD40 structure (Zhang et al., 2019), and now the RCKW structure (Deniston et al., 2020). However, except for two very low-resolution structures (Guaitoli et al., 2016; Sejwal et al., 2017), nothing definitive was known about the kinase domain nor about the interactions of the kinase and GTPase domains (Roc). Furthermore, while we have hundreds of kinase structures in the literature, most represent the kinase domain only and many are in the presence of nucleotides and/or inhibitors and shed little light on peptide recognition or on the important ways in which the kinase is allosterically regulated, either positively or negatively, by its flanking domains. These critical aspects can now be addressed for the first time for LRRK2 that a relatively high-resolution (3.5 Å) cryo-EM structure of a monomeric RCKW domain in an inactive conformation is available (Deniston et al., 2020). This structure captures the four C-terminal domains including both catalytic domains. Recent structures of BRaf also highlight how important it is to look at full-length proteins and protein complexes (Kondo et al., 2019; Park et al., 2019; Liau et al., 2020). Thus the recent Cryo-Electron Tomography (cryo-ET) structure showing helical polymers of a full-length dimeric LRRK2 mutant (I2020T) wrapped in a closed and active conformation around microtubules allows us to further appreciate the complexity of the domain organization and in particular how the release of the N-terminal domains exposes the C-terminal RCKW domain (Watanabe et al., 2020). Based on these structures and our earlier analysis of the LRRK2 kinase domain (Schmidt et al., 2019), we describe here some of the novel features of the LRRK2 kinase domain and compare it to PKA, Src, and BRaf. These features include the hydrophobic spine architecture, the αC-β4 Loop, and the Activation Segment of the LRRK2 kinase domain (Figures 1B,C) Such a comparison of LRRK2’s kinase domain with other well-understood kinases provides fundamental insight to its activation/regulation and nucleotide-binding features. Also, a comparative analysis of the G-Loop in the kinase domain and the P-Loop in the ROC/GTPase domain, the two most important nucleotide-binding motifs in biology, is presented. A general model of the active kinase domain of LRRK2 showing the alignment of the R-Spine as well as the sequence alignment for the four kinases is included as a frame of reference in Figure 1D. Overall, our analyses provide a dynamic portrait that shows how the N- and C-lobes of the kinase domain create a central allosteric hub that drives the dynamic transitions that LRRK2 undergoes as it toggles between its active and inactive states.

### Activation of LRRK2

Protein kinases are highly dynamic molecular switches that are tightly regulated both in their activation and localization. In the case of LRRK2, it also shuttles between monomeric and oligomeric states, and multiple 14–3–3 binding sites have been identified that likely stabilize these distinct conformational states through intramolecular and/or intermolecular mechanisms similar to what was recently revealed for BRaf (Kondo et al., 2019; Park et al., 2019; Liau et al., 2020). The conserved kinase core is defined not only to be a set of highly conserved residues that mediate substrate and nucleotide-binding and phosphoryl transfer but also by a highly conserved hydrophobic core that provides a dynamic scaffold for allosteric regulation of catalysis and activation. The Regulatory (R) and Catalytic (C) Spines anchored to the hydrophobic αF-helix that spans the C-lobe define the core architecture of every protein kinase (Kornev et al., 2008; Taylor and Kornev, 2011), and the assembled R-spine is the hallmark signature motif of every active protein kinase (Kornev et al., 2006; Kornev and Taylor, 2015). The intrinsic switch mechanism that leads to the activation of every kinase is embedded in the assembly of the R-spine. Here we will focus first on the LRRK2 R-spine and how it is dynamically assembled as a consequence of kinase inhibitor binding and in response to selective PD mutations.

### Regulatory and Catalytic Spines of LRRK2

**Figure 2:**
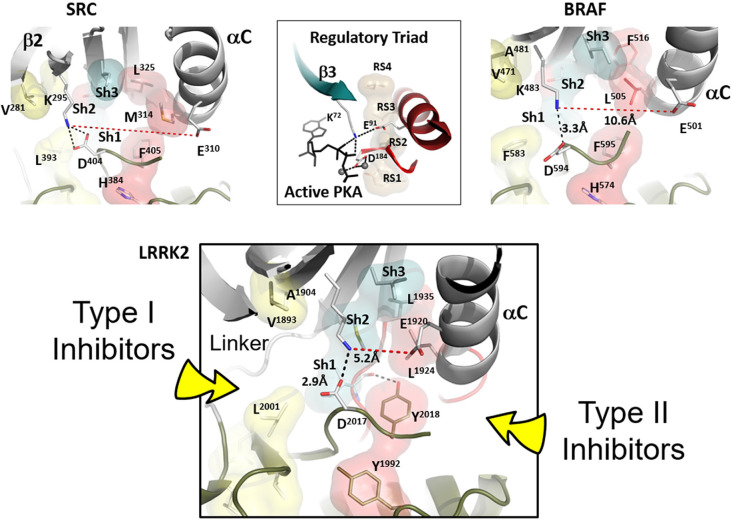
Confirmation of Inactive LRRK2 is compared to inactive BRaf and Src. The conformation of inactive LRRK2 as seen in the recent RCKW structure (Deniston et al., 2020) is at the bottom panel. For comparison, an inactive conformation of Src and BRaf are shown on the top panel with the shells of the R-spine residues in red and the Shell residues in teal. In the middle of the top panel is the active conformation of the regulatory triad of PKA when it is bound to ATP and Mg ions. The aligned R-spine in active PKA is indicated by beige shells. The bottom panel depicts LRRK2 in an inactive conformation (Deniston et al., 2020). The arrow on the left indicates type I protein kinase inhibitor specific interactions with the linker/hinge region, while the right arrow points to the deep pocket only accessible in the DFG “out” conformation. Type II protein kinase inhibitors protrude into this pocket to stabilize the DFG “out” conformation. Both inhibitor types are ATP competitive (Röhm et al., 2020).

The R-spine consists of four residues referred to as RS1, RS2, RS3, and RS4. The essential features of the broken and inactive R-spine in LRRK2 are defined in Figure 2 and compared to two inactive kinases, Src (Figure 2, top left) and BRaf (Figure 2, top right). PKA is used as a frame of reference for the conserved hallmarks of an active kinase where the R-spine is assembled. In this active kinase conformation, the four R-spine residues through hydrophobic contacts interact with each other forming an extended motif that connects the N- and C-lobes of the kinase core. This creates an active conformation that correctly orients the DFG motif, the αC-helix, and the activation loop, all needed for MgATP binding and phosphoryl-transfer (Kornev et al., 2006; Kornev and Taylor, 2015). In contrast, how the R-spine can be broken is not conserved as is demonstrated nicely with these three kinases (BRaf, Src, and LRRK2). The RS3 residue, L1924 in LRRK2, is embedded in the αC-helix, and this helix is in an “out” conformation when the R-spine is broken in LRRK2, BRaf, and Src. There are also three key conserved regulatory residues, referred to as a “Regulatory Triad” that are assembled in a very precise way in every active kinase. These three residues provide the correct positioning of ATP and two Mg^2+^ ions (Figure 2). In LRRK2 these are K1906 and E1920 in the N-Lobe and D2017 in the DFGѱ, motif of the C-Lobe. K1906 is in β-strand 3 and is part of the G-Loop motif discussed later while E1920 is part of the αC-Helix. The numbering of the key residues from Src, BRaf, PKA, and LRRK2 are provided (Supplementary Table 1).

In addition to the R-spine residues, there are two highly conserved Catalytic (C)-Spine residues in the N-Lobe (V1893 and A1904 in LRRK2), and these residues provide a hydrophobic cap for the buried adenine ring of ATP. There are also three “Shell” residues in the N-lobe that contribute to the hydrophobic core architecture (Meharena et al., 2013); Sh1 is I1933, Sh2 is M1947, and Sh3 is L1945. These shell residues lie between the two spines. M1947 is the “Gatekeeper” residue (Okuzumi et al., 2009) and bridges β-strand 5 with the short linker that joins the N- and C-lobes. The LRRK2 “gatekeeper” mutant was used to identify new substrates using chemical genetic analysis (Krumova et al., 2015). I1933 is the only shell residue that directly links the R- and C-spines, and this Isoleucine also interacts with the γ-phosphate of ATP. It provides a hydrophobic docking surface for the phosphate and for the electrostatic bridge between K1906 and E1920 that is also a characteristic feature of the closed conformation. Mutating this Sh1 Valine to Glycine in PKA inactivates the kinase (Meharena et al., 2013). These three shell residues collectively contribute significantly to the hydrophobic architecture of the kinase core, and V104 (I1933 in LRRK2) is localized specifically in the middle of the αC-β4 loop as discussed below.

LRRK2 in the RCKW structure is in an open and inactive conformation (Deniston et al., 2020), and this structure is compared to BRaf and Src in Figure 2. All three kinases in this figure are in an inactive conformation. Clearly, the inactive conformation in each of these kinases is different, but in all cases, the R-spine is broken. How the open and inactive conformation of LRRK2 is stabilized is especially noteworthy and interesting in that it explains our earlier observation that relates to the DFGѱ motif. In most other kinases this highly conserved motif is DFGѱ, and the Phenylalanine is an R-spine residue, RS2. In LRRK2 this Phenylalanine is a Tyrosine, and we discovered that mutating this Tyrosine to Phenylalanine leads to constitutive activation of LRRK2; this mutant also docks spontaneously onto microtubules (Schmidt et al., 2019). We thus hypothesized that this Tyrosine serves as a “brake” to keep LRRK2 in an open and inactive state. The RCKW structure confirms this hypothesis (Deniston et al., 2020). As seen in Figure 2, the side chain hydroxyl group of Y2018 is firmly anchored to the backbone amide and carbonyl of I1933, which is the Sh1 residue, ensuring that both R-spine residues in the N-lobe are locked into an inactive conformation. Besides, the misalignment of the Regulatory Triad will also be stabilized. Simply removing that single hydroxyl moiety would allow the kinase domain to favor an active conformation that can dock onto microtubules even in the absence of the highly specific LRRK2 type I inhibitor MLi-2. This is similar to the I2020T mutant but distinct from the G2019S mutant, which is also constitutively active, like the Y2018F mutant, but still requires MLi-2 to bind to microtubules. Clearly, the DFGѱ motif is a hot spot for allosteric regulation, and it is, in general, the most highly mutated region in other kinases where mutations lead to the creation of oncogenes (Torkamani et al., 2008).

Type I kinase inhibitors such as MLi-2 favor an active DFG “in” conformation, where the R-spine is assembled (Figure 2, bottom panel), whereas type II kinase inhibitors favor a DFG “out” conformation (Röhm et al., 2020). The active conformation of LRRK2 is reflected in the Cryo-ET structure of full-length LRRK2 (Watanabe et al., 2020). Deniston et al. (2020) show that type II inhibitors prevent docking to microtubules. Type II inhibitors target the DFG “out” conformation and this will be a quiet variable in each kinase. In Figure 2 (bottom panel) we point out the general region that will be targeted by type I and type II inhibitors.

## Conserved αC-β4 Loop Is A Hub for Structure, Function, and Protein: Protein Interactions

**Figure 3:**
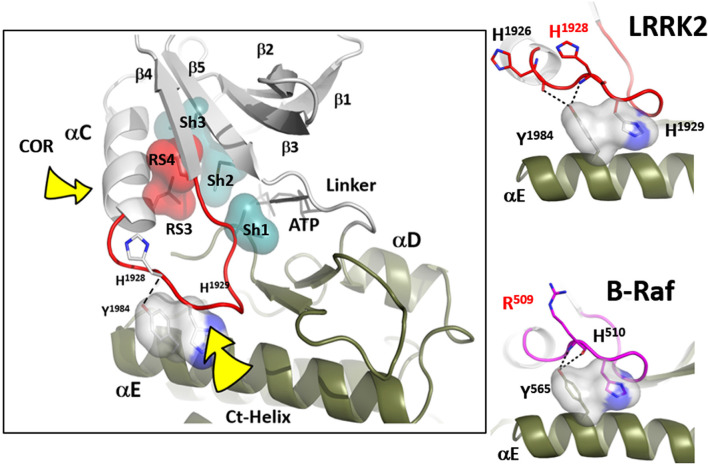
The αC-β4 loop of LRRK2. The highly conserved αC-β4 loop of LRRK2 is indicated in red. The two hydrophobic R-spine residues are shown as a red transparent surface while the Sh1 residue that touches the adenine ring of ATP is in teal. Sh2 and Sh3 also in teal serve as a further hydrophobic bridge. RS3 (L1924) is at the N-terminus of the αC-β4 Lop while the C-terminus is the RS4 (L1935) residue. In the active conformation RS3 and RS4 are aligned with RS1 and RS2 in the C-Lobe. RS4 is always firmly anchored to the β-sheet while RS3 lies at the C-terminus of the aC-Helix which can flip “in” and “out.” The two right panels show how the αC-β4 Loop is anchored to the αE-helix.

Another essential but less appreciated conserved part of the kinase active site is the αC-β4 Loop that spans the C-terminus of the αC-Helix and β strand 4. This loop in PKA moves as a rigid body with the C-lobe (Tsigelny et al., 1999), and two of the R-spine residues as well as the Sh1 residue are embedded in the αC-β4 loop (Figure 3). The tip of the αC-β4 loop which includes the side chain of H1929 and the backbone of H1928 in LRRK2 is the only piece of the N-lobe that is always anchored to the C-lobe when one considers the rigid body movements of PKA. While the G-loop between β1 and β2 is highly flexible and the αC-helix can move in or out as part of the mechanism for assembling the R-spine, the β-sheet and the αC-β4 loop remain fixed; they are not flexible. The αC-β4 loop is flanked by the two R-spine residues in the N-lobe, RS3, or L1924 that lies one turn of the helix beyond E1920 and RS4 or L1935 that marks the beginning of β4. RS4 is always anchored firmly to the five-stranded beta-sheet that spans the N-lobe while RS3 toggles in and out as a function of the assembly of the R-spine (Taylor et al., 2019). Another important interaction of the αC-β4 loop with the C-lobe is mediated by a highly conserved Tyrosine in the αE-helix. The hydroxyl moiety of this Tyrosine binds to the backbone of H1928 in the αC-β4 loop and is also a critical part of this motif. How the αC-β4 Loop is anchored by a conserved Tyrosine in the αE-helix of both Src and BRaf is also highlighted in Figure 3.

While the tip of the αC-β4 loop and the backbone of H1928 are anchored to the C-lobe, the flanking regions of this motif often interact with elements that lie outside the kinase core. It is a “hot spot” for protein:protein interactions. Thompson et al. (2009) defined a set of spatially conserved pockets on the surface of the kinase core although the chemical properties of these pockets were variable. Perhaps the best studied of these surface pockets is the hydrophobic PIF pocket in the AGC kinases, including PKA, which binds to and stabilizes the αC-helix (Biondi et al., 2000; Hindie et al., 2009). In LRRK2 this region appears to be stabilized by the COR domain, which is confirmed by hydrogen-deuterium exchange/mass spectrometry (manuscript in preparation). The combined αC-helix/αC-β4 loop is a critical allosteric docking site for all protein kinases.

**Figure 4:**
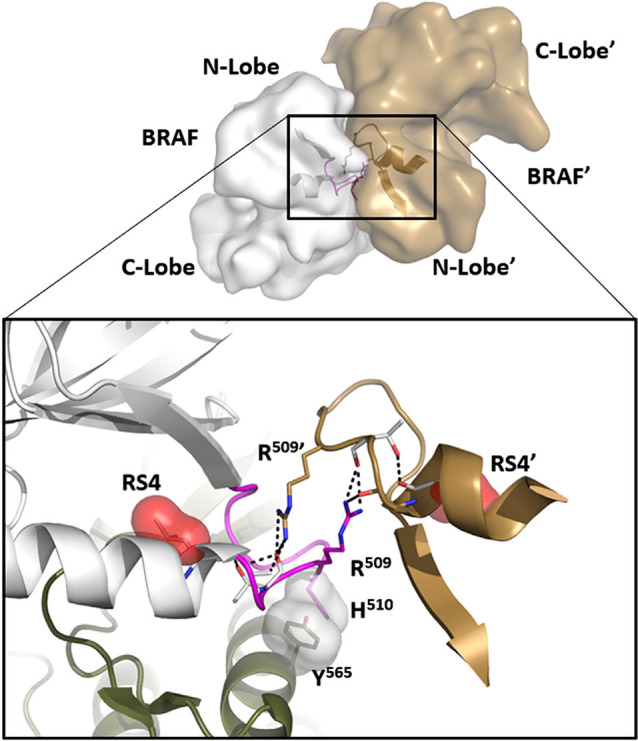
The αC-β4 Loop in BRaf is a dimer interface. At the top is the anti-parallel BRaf dimer. The close-up view of this interface (bottom) shows how R509 in the αC-β4 Loop drives dimer formation and mutating this Arginine breaks the dimer. R509 in BRaf is homologous to H1928 in LRRK2. Another key interaction that is conserved in all kinases is the anchoring of the αC-β4Loop to the αE-Helix through a conserved hydrophobic Tyr or Phe (Y565).

**Figure 5:**
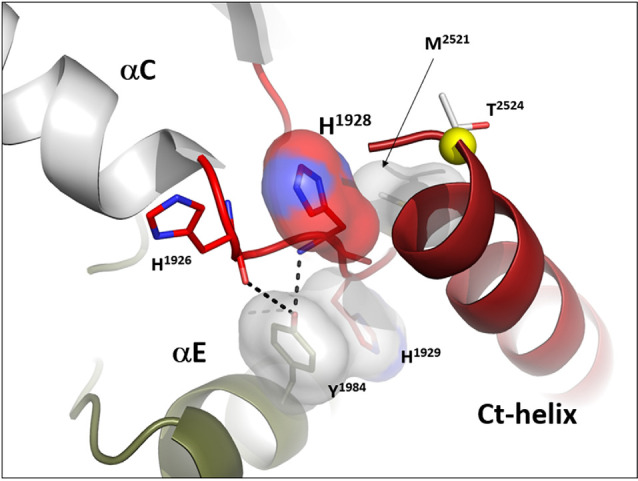
The αC-β4 Loop in LRRK2 is an interface between the C-terminal helix that follows the WD 40 domain. The αC-β4 Loop of LRRK2 is also anchored to the αE-Helix through a conserved Y1984 in αE and H1929 at the tip of the αC-β4Loop. H1928, analogous to R509 in BRaf is part of the docking interface for the Ct-helix. T2524 is a putative docking site for a 14-3-3 (Manschwetus et al., 2020).

In many ways, we can think of these combined motifs, the αC-Helix, and the αC-β4-Loop, as bi-functional. One surface of the αC-helix contains conserved residues that contribute to the active site while the other surface is facing away from the active site and is controlled by the tails that flank the kinase core or by other proteins that regulate the position of the helix. In the same way, one surface of the αC-β4 loop faces the active site where the γ-phosphate of ATP is located, while the other surface is known to be a potential allosteric docking surface. The surface facing the active site cleft is conserved across the kinome; it provides a platform for the catalytic residues and mediates interactions that are shared by all protein kinases. In contrast, the other faces solvent and is variable; it provides an allosteric surface that can be regulated by many interacting domains and proteins. In the case of BRaf, this surface provides the asymmetric interface in the BRaf dimer (Figure 4). R509, in particular, that is stabilized by backbone interactions with Y565 in the αE-helix (Figure 3) is a critical part of this dimer interface and dimerization is thought to be an important part of the activation mechanism for BRaf (Hu et al., 2013). Many oncogenic mutations in BRaf, including a mutation that enhances the hydrophobicity of the RS3 residue, “hijack” this finely tuned regulatory mechanism and lead to constitutive activation that is now independent of Ras-mediated dimerization (Hu et al., 2013; Shaw et al., 2014). In the case of LRRK2, this important allosteric surface is a docking site for the C-terminal helix that follows the WD40 domain (Figure 5). This helix spans both lobes of the kinase core, and the C-terminal residues are docked firmly onto the αC-β4 Loop in the inactive conformation (Deniston et al., 2020). This C-terminal helix will certainly be an important motif for the regulation of the kinase domain of LRRK2 and, based on cross-linking experiments with inactive LRRK2, will likely also be an important interacting surface for the ARM:ANK:LRR repeats (Guaitoli et al., 2016). Finally, and perhaps most intriguing, is the phosphorylation site that lies at the very end of the C-terminal helix (Pungaliya et al., 2010). Most recently Manschwetus et al. have identified pT2524 as a putative 14–3–3 binding site (Manschwetus et al., 2020).

## Activation Loop

**Figure 6:**
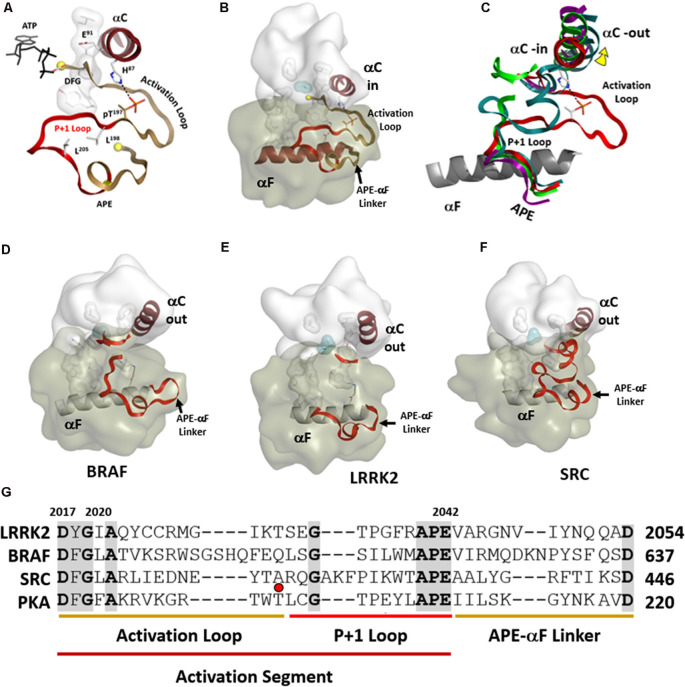
Activation segments. **(A)** The Activation Loop (tan) and the P+1 Loop (red) in their active conformation, as exemplified by PKA, are shown on the top left. **(B)** The motif that links the activation segment to the αF-helix (also shown in tan) provides an allosteric docking surface for substrates and other regulatory proteins. These regions are highlighted at the bottom in the sequence alignment of PKA, LRRK2, BRaf, and Src. **(C)** The three activation segments are aligned and compared to active PKA shown on the right. In each of these inactive structures **(D–F)**, the αC-helix is in an “out” conformation. The middle panel shows the different ways in which the Activation segment is ordered or disordered in inactive conformations of Src, BRaf, and LRRK2. **(G)** These regions are highlighted at the bottom in the sequence alignment of PKA, LRRK2, BRaf, and Src. The red dot corresponds to the phosphorylation site in the activation loop of PKA.

Kinases are dynamically assembled in ways that often involve the Activation Loop (AL) which in most kinases contains a key phosphorylation site (Johnson and Lewis, 2001; Nolen et al., 2004). This entire region that includes the Activation Loop and the P + 1 Loop is referred to as the Activation Segment (Figure 6). The segment is flanked on the N-terminal side by the DFGѱ motif while it is flanked at the other end by the APE motif (Figure 6G). The APE motif at the C-terminus of the P + 1 Loop is anchored to the αF-helix, and this node (APE-αF Linker) provides an allosteric docking site for many substrates and inhibitor proteins such as PKI and PKA regulatory subunits (Knighton et al., 1991; Johnson et al., 2001; Kim et al., 2005; Figures 6A,B). In many kinase structures, perhaps most, the AL is disordered. Most likely this is because we typically look at only the kinase domain and not the full-length protein, and in the absence of phosphorylation the kinase is not fully active. When the kinase is in an inactive state this loop can be ordered by other parts of the protein or by other interacting proteins. The RS2 R-spine residue is embedded in the DFGѱ-motif of the AL and many inactive kinases have a DFG “out” conformation. How this region is ordered in inactive full-length protein kinases is a major question that is mostly still unknown. The structure of full-length BRaf gave us some clues for the first time about the ordering of the AL when BRaf is in an inactive conformation (Kondo et al., 2019; Park et al., 2019; Liau et al., 2020).

In the open and inactive conformation of LRRK2, as seen in the recent RCKW structure (Deniston et al., 2020), the AL as well as most of the P + 1 Loop are disordered (Figure 6E). In inactive Src the entire Activation Segment is ordered in a nonactive conformation (Xu et al., 1999; PDBID:2SRC). In BRaf (Ren et al., 2012; PDBID:4E4X) the P + 1 Loop is partially ordered but in an inactive conformation, and most of the AL is disordered (Figure 6D). These structures suggest that the P + 1 Loop may, in general, be more flexible than we have previously assumed. In the case of the RCKW structure, which is also in contrast to most other kinase structures, there is no nucleotide bound, and this could also contribute to the altered conformation of the P + 1 loop. There are three potential phosphorylation sites in the AL of LRRK2 and two have been identified as functional sites (Greggio et al., 2008; Li et al., 2010). Most likely, based on cross-linking experiments with full-length LRRK2, the AL in full-length inactive LRRK2 is ordered by regions that are embedded in the ANK/ARM/LRR repeats (Guaitoli et al., 2016) but this is another of the important questions that need to be resolved. Greggio et al. (2008) demonstrated that an N-terminal truncation construct of LRRK2 which lacks the Ank, Arm, and LRR repeats exhibits enhanced kinase activity. This points out a crucial inhibitory function of the N-terminus of LRRK2 and indicates that the observed increased kinase activity by many of the PD mutations is most likely initiated by “unleashing” the N-terminal domains. This regulation by the N-terminus of LRRK2 is analogous in many ways to BRaf where so many oncogenic mutations release the N-terminal Ras-binding domain and expose the kinase domain (Hu et al., 2011, 2013, 2015). LRRK2 and BRaf belong to the same branch of the Kinome tree so most likely there are many lessons that we can learn from BRaf that will apply to LRRK2.

Although the recent apo RCKW structure represents an open and inactive conformation, Deniston et al. (2020), in collaboration with Watanabe et al. (2020), show that occupancy of the ATP binding site in the kinase domain is a critical switch that controls the conformation of RCKW. They show, in particular, that type I inhibitors that canonically occupy the Adenine binding pocket generate a closed conformation. This closed conformation is similar to the MLi-2 bound structure of a humanized *Dictyostelium* Roco4 kinase (Gilsbach et al., 2015), and it is this closed conformation that is capable of forming long polymers that can dock onto microtubules as seen in the Villa structure (Watanabe et al., 2020). It is known that wild-type full-length LRRK2 does not dock onto microtubules spontaneously but rather shows a cytosolic distribution in cells; however, when treated with a type I kinase inhibitor such as MLi-2 or LRRK2in1, the microtubules become decorated with LRRK2 polymers (Deng et al., 2011; Blanca Ramírez et al., 2017; Leandrou et al., 2019; Schmidt et al., 2019). This correlates with a closed conformation, and three of the four common mutations also appear to induce a closed conformation where full-length LRRK2 docks spontaneously onto microtubules (Kett et al., 2012). The kinase-dead mutants do not dock onto microtubules even in the presence of MLi-2 suggesting either that they are not capable of forming a fully “closed” conformation or are not able to bind MLi-2 (Schmidt et al., 2019). While occupancy of the ROC/GTPase domain with nucleotide is likely to also be a conformational sensor (Wauters et al., 2018; Deyaert et al., 2019), it is the opening and closing of the kinase domain that appears to be the major driver of conformational changes in LRRK2. While much future work is needed to decipher the mechanisms that allow these two switch domains to communicate with each other and with the rest of the molecule, it is important here to elucidate the fundamental differences between a P-loop and a G-Loop and in particular to compare and contrast the specific P-loop and G-Loop in LRRK2.

## P-Loops and G-Loops Provide Distinct Mechanisms for Nucleotides to Regulate LRRK2

**Figure 7:**
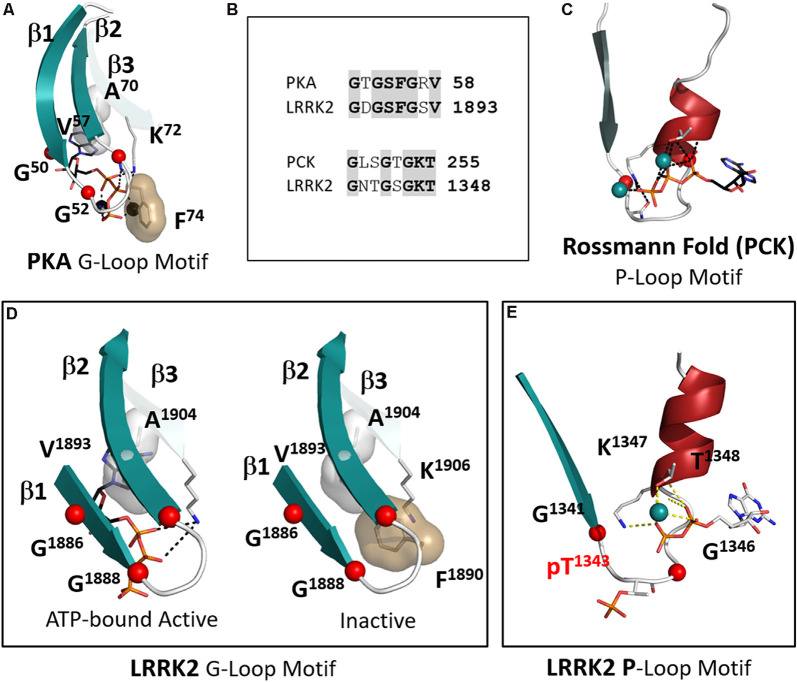
The P-Loop and G-Loop of LRRK2. **(A)** The canonical G-Loop found in all kinase domains. The adenine ring is buried under the first three β-strands with Alanine in β-strand 3. Valine in β strand 2 being highly conserved C-spine residues that cap the upper surface of the adenine ring. **(B)** The sequences of the G-Loop in PKA and LRRK2 are aligned at the top and the sequences of the P-Loop of phosphoenolpyruvate carboxykinase (PCK) and LRRK2 are aligned at the bottom. **(C)** The canonical P-loop first described by Rossmann (Ramakrishnan et al., 2002). Although it is also associated with ATP binding the architecture of this loop is distinct from the G-Loop. The Glycine-rich loop is preceded by a beta-strand as in the G-Loop, but it is then followed by a helix that contains the conserved Lysine. The nucleotide is positioned typically on the surface of the protein while the γ-phosphate of ATP is buried under the loop. **(D)** The G-Loop of LRRK2 conforms to this canonical architecture. Two conformations are shown in the box: on the left is a model with ATP-bound; on the right is the apo structure (Deniston et al., 2020). **(E)** LRRK2 has a canonical P-loop in the ROC domain that binds to GTP/GDP. In the RCKW structure, GDP is bound and the loop contains a phosphorylated Threonine (T1343). The conserved glycines are shown as red balls.

LRRK2 is highly unusual in that it has both a P-Loop in the ROC/GTPase domain and a classic G-Loop in the kinase domain. Binding of nucleotides is the key mechanism that allows both of these switches to function and it is important to appreciate the distinction between them (Saraste et al., 1990; Kornev and Taylor, 2015). With LRRK2 we have the unique opportunity to observe a P-loop and a G-Loop in the same molecule. In this first 3.5 Å structure of the RCKW domain GDP(Mg) is bound to the ROC/GTPase domain while the kinase domain is in its apo state. Although both motifs contain a glycine-rich loop and a conserved Lysine and both bind nucleotides and metal ions (Figures 7A–C), the mechanisms by which they bind their nucleotides are fundamentally different. The P-Loop (also known as the “Walker motif”) belongs to the classical family referred to as the “Rossman fold” and consists of a β-strand followed by a glycine-rich loop and a helix where the conserved Lysine is located (Figure 7C; Ramakrishnan et al., 2002). Until the first protein kinase structure of PKA was solved it was assumed that all nucleotide-binding sites would conform to the “Rossman Fold.” In the case of the P-loop, the adenine ring is more solvent-exposed and the γ-phosphate is at the base of the cleft so that closing of the cleft, as in the case of hexokinase, brings the substrate close to the γ-phosphate (Ramakrishnan et al., 2002). In the case of ATPases and GTPases, the γ-phosphate is transferred to the water. With the ATPase-driven motors, we see how exquisitely sensitive these loops are to the state of the nucleotide (Vale and Milligan, 2000; Lyubimov et al., 2011). In the case of the G-Loops in protein kinases, the adenine ring is buried at the base of the cleft while the γ-phosphate is pointing outwards towards the catalytic loop and the R-spine (Figure 7A). The closing of the active site cleft fuses the two parts of the C-spine that come from the N- and C-lobes and this buries the adenine ring in a mostly hydrophobic shell. The G-Loop also begins with a β-strand followed by a glycine-rich loop but then it is followed by two more β-strands. Each of these strands has a critical and highly conserved hydrophobic residue that caps the “top” of the adenine ring of ATP while the third β-strand also contains the conserved Lysine that binds to the ATP phosphates. In LRRK2 these hydrophobic C-spine residues are V1893 in β2 and A1904 in β3.

Another key hydrophobic residue in the G-Loop immediately precedes the third Glycine (Figure 7B). This residue is usually a Phenylalanine or a Tyrosine and when the kinase is in an active conformation and bound to ATP this residue shields the γ-phosphate of ATP from the solvent. In the inactive RCKW structure, F1890 is folded under β strands 1 and 2. This location is unusual but has been observed in several other kinases when nucleotide or inhibitor is missing. Most likely in the absence of nucleotide, this Phenylalanine is flexible (Figure 7D). Not many structures are available that lack nucleotide, but it is intriguing to hypothesize that binding of nucleotide forces this side chain into an “out” conformation where it is now “primed” to bind substrate and guide the transfer of the phosphate.

The P-Loop in the ROC/GTPase domain corresponds to “Switch I” in the GTPase terminology (Yao et al., 2016). In the RCKW structure, this site is occupied by GDP (Figure 7E). However, in the RCKW structure, there is another unusual feature that has not been observed or commented on in other GTPase structures. There is a single phosphate in the RCKW structure, pT1343, and it is located precisely in the middle of the G-loop. If we compare many other GTPase sequences, including the highly homologous *Dicytostelium* and *C. tepidium* ROC:COR domains from the LRRK2 homologs, there is no Threonine or Serine. Instead, this position is preceded by an acidic residue, and this acidic residue is conserved in many GTPase domains. The Threonine in LRRK2 may be a feature of the more highly evolved mammalian LRRK2 structures. While the biological significance of this Threonine remains to be determined, it is positioned in a strategically important region. The donor of this phosphate may be the GTP that is bound to the ROC domain. There would also likely be functional consequences of this phosphorylation event. This phosphorylated form of RCKW could not, for example, form the dimer that is seen in the earlier ROC:COR structures. A similar phosphorylated residue has not been previously reported in other GTPases so it could be highly dynamic and not usually trapped. It is perhaps a unique feature of cryo-EM that allows one to trap different conformational states that might otherwise be washed out by averaging in a crystal structure.

### The Kinase Domain Is the Driver of LRRK2 Dynamics

With structures now in hand, we are poised to explore some of the detailed mechanisms that allow LRRK2 to toggle between its active and inactive states and most importantly to understand how PD mutations interfere with this finely tuned regulatory switch. How do multiple phosphorylation sites as well as nucleotide-binding influence the structure and function of LRRK2? How does the binding of 14–3–3 influence the conformation, activity, and localization of LRRK2? and most importantly how do PD mutations disrupt the normal finely tuned functioning and lead to pathogenic states? These are our next exciting challenges. From these first publications of human LRRK2 structures, however, it is already clear that the kinase domain will be a major driver of these conformational transitions. It is also clear that the N-Lobe of the kinase domain will be regulated not only by its hydrophobic core and by nucleotide-binding but also by the domains that flank it. The CORB domain will influence the αC-Helix while the C-terminal helix will communicate with the C-lobe and the αC-β4 loop. Most intriguingly in this structure, we see for the first time how the activation loop of a kinase comes close to a GTPase domain. We also see, how key phosphorylation sites, strategically positioned around the kinase core, are poised to influence the structure, function, and cellular location of LRRK2.

## Author Contributions

Our thinking about the kinase domain of LRRK2, as summarized in this review article, is based on our collective discussions and deliberations over the past year. It is based on our knowledge of the activation of other kinases and our analysis of the cryoEM structure of the RCKW domain, which provides for the first time a template for human LRRK2. ST and PK-S contributed to writing the manuscript while J-HW, PA, SS, and FH edited the drafts and the revisions. SK and SM not only reviewed and edited the manuscript but also provided purified RCKW protein to Leschziner and his colleagues for the cryoEM work. This protein led to the eventual structure solution by CryoEM. All authors contributed to the article and approved the submitted version.

## Conflict of Interest

The authors declare that the research was conducted in the absence of any commercial or financial relationships that could be construed as a potential conflict of interest.
