# Supplementary material for: Kinase Domain Is a Dynamic Hub for Driving LRRK2 Allostery
Source: Front Mol Neurosci. 2020 Oct 6;13:538219. doi: 10.3389/fnmol.2020.538219 (PMC7573214; doi:10.3389/fnmol.2020.538219)
Supplement: Supplementary file 1 [file Table_1.DOCX]

**Supplement**

**Kinase domain is a dynamic hub for driving LRRK2 allostery**

**Susan Taylor1*, Pallavi Kaila-Sharma1, Jui-Hung Weng1, Phillip Aoto1, Sven Schmidt2, Stefan Knapp3, Sebastian Mathea3, Friedrich W. Herberg2**

1University of California, San Diego, United States, 2University of Kassel, Germany, 3Goethe University Frankfurt, Germany

**Supplement Table 1: Comparison of important residues for function and regulation of model kinases and LRRK2.**

| **Motif and numbering** | **PKA** | **LRRK** | **BRaf** | **SRC** |
| --- | --- | --- | --- | --- |
| **C-spine** | | | | |
| CS8 | A70 | A1904 | A481 | A293 |
| CS7 | V57 | V1893 | V471 | V281 |
| CS6 | L173 | L2001 | F583 | L393 |
| CS5 | I174 | L2002 | L584 | V394 |
| CS4 | L172 | V2000 | I582 | I392 |
| CS3 | M128 | L1955 | L537 | L346 |
| CS2 | M231 | I2066 | L649 | L455 |
| CS1 | L227 | L2062 | V645 | L451 |
| **R-spine** | | | | |
| RS4 | L106 | L1935 | F516 | L325 |
| RS3 | L95 | L1924 | L505 | M314 |
| RS2 | F185 | Y2018 | F595 | F405 |
| RS1 | Y164 | Y1992 | H574 | H384 |
| **Shell** | | | | |
| Sh3 | M118 | L1945 | I527 | I336 |
| Sh2 | M120 | M1947 | T529 | T338 |
| Sh1 | V104 | I1933 | L514 | V323 |
| **Regulatory triad** | | | | |
| RT3 | D184 | D2017 | D594 | D404 |
| RT2 | E91 | E1920 | E501 | E310 |
| RT1 | K72 | K1906 | K483 | K295 |
